# Policy-makers’ perspectives on implementation of cross-sectoral nutrition policies, Western Pacific Region

**DOI:** 10.2471/BLT.20.283366

**Published:** 2021-09-29

**Authors:** Erica Reeve, Anne-Marie Thow, Oliver Huse, Colin Bell, Anna Peeters, Gary Sacks

**Affiliations:** a Institute for Health Transformation, Deakin University, 221 Burwood Highway, Burwood, VIC 3125, Australia.; b Menzies Centre for Health Policy, University of Sydney, Sydney, Australia.

## Abstract

Implementation of effective cross-sectoral nutrition policies remains a challenge worldwide. By reviewing reports from World Health Organization meetings and consultations – convened for policy-makers representing Member States of the Western Pacific Region – we provide an insight into how national policy-makers and external actors can support different dimensions of nutrition policy implementation. Key insights of policy-makers attending food and nutrition-centred meetings include that country-level implementation of nutrition policy relies on strong policy design, organizational planning and governance mechanisms that promote collective responsibility across multiple sectors. Policy-makers responsible for implementing nutrition policies face major challenges resulting from limited capacity, both within and external to government, particularly in relation to monitoring and enforcement activities. Successful implementation of nutrition policy measures will require greater political will to provide the requisite resources and institutional structures to ensure sustained policy effectiveness. Nongovernmental partners, including international agencies and researchers, have an opportunity to support policy implementation by providing technical support to Member States to frame action on nutrition in a more compelling way. They can also help policy-makers to build the organizational and structural capacity to coordinate cross-sectoral policy. Improved policy design, planning and governance and strategic capacity-building, supported by external partners, can strengthen the sustained implementation of cross-sectoral nutrition policy and improve nutrition outcomes.

## Résumé

Le déploiement de mesures nutritionnelles intersectorielles efficaces reste un défi à travers le monde. En examinant des rapports de réunions et consultations convoquées par l'Organisation mondiale de la Santé (OMS) pour les représentants des États Membres de la Région du Pacifique occidental, nous analysons la manière dont les responsables politiques nationaux et les acteurs externes peuvent y contribuer à différents niveaux. Parmi les principaux enseignements tirés des responsables ayant participé à des réunions sur l'alimentation et la nutrition, citons la nécessité d'élaborer une politique solide pour appliquer des mesures nutritionnelles à l'échelle nationale, la planification organisationnelle et les mécanismes de gouvernance qui font appel à la responsabilité collective dans de multiples secteurs. Les législateurs chargés d'appliquer ces mesures nutritionnelles sont confrontés à des défis majeurs car ils disposent de capacités limitées, tant au sein du gouvernement qu'en dehors, en particulier lorsqu'il s'agit d'activités de surveillance et d'exécution. Pour que la mise en œuvre de telles mesures soit couronnée de succès, il faut consolider la volonté politique afin de fournir les ressources et les structures institutionnelles requises pour garantir une efficacité durable. Les partenaires non gouvernementaux, notamment les chercheurs et les organismes internationaux, peuvent soutenir cette mise en œuvre en apportant une assistance technique aux États Membres, dans le but d'encadrer les mesures nutritionnelles de façon plus convaincante. Ils peuvent également aider les législateurs à développer les capacités structurelles et organisationnelles leur permettant de coordonner les politiques intersectorielles. Améliorer la gouvernance, la planification, l'élaboration de politiques et la création de capacités stratégiques avec le concours de partenaires externes peut renforcer l'application à long terme de politiques nutritionnelles intersectorielles et générer de meilleurs résultats en matière de nutrition.

## Resumen

La aplicación de políticas intersectoriales de nutrición efectivas sigue siendo un desafío a nivel mundial. Mediante la revisión de los informes de las reuniones y consultas de la Organización Mundial de la Salud (OMS), dirigidas a los responsables de formular políticas que representan a los Estados miembros de la región del Pacífico Occidental, se presenta una reflexión sobre cómo los responsables de formular las políticas nacionales y los agentes externos pueden apoyar las diferentes dimensiones de la aplicación de las políticas de nutrición. Entre los principales puntos de vista de los responsables de formular políticas que asistieron a las reuniones sobre alimentación y nutrición figura la idea de que la aplicación de las políticas de nutrición a nivel nacional depende de un diseño sólido de las políticas, de la planificación organizativa y de los mecanismos de gobernanza que promueven la responsabilidad colectiva en múltiples sectores. Las autoridades responsables de aplicar las políticas de nutrición se enfrentan a importantes retos derivados de la limitada capacidad, tanto dentro como fuera del gobierno, sobre todo en relación con las actividades de seguimiento y cumplimiento. El éxito de la aplicación de las medidas políticas sobre nutrición requerirá una mayor voluntad política para suministrar los recursos necesarios y las estructuras institucionales que garanticen la efectividad sostenida de las políticas. Los asociados no gubernamentales, incluidos los organismos internacionales y los investigadores, tienen la oportunidad de apoyar la aplicación de las políticas al proporcionar apoyo técnico a los Estados miembros para estructurar las medidas sobre nutrición de manera más convincente. También pueden ayudar a los responsables de formular políticas a crear la capacidad organizativa y estructural necesaria para coordinar las políticas intersectoriales. La mejora del diseño, la planificación y la gobernanza de las políticas, así como la creación de capacidades estratégicas, con el apoyo de asociados externos, pueden reforzar la aplicación sostenida de las políticas intersectoriales de nutrición para mejorar sus resultados.

## ملخص

لا يزال تنفيذ سياسات التغذية الفعالة عبر عدة قطاعات تحديًا في جميع أنحاء العالم. من خلال مراجعة التقارير الواردة من اجتماعات ومشاورات منظمة الصحة العالمية (WHO)، التي انعقدت لواضعي السياسات الذين يمثلون الدول الأعضاء في منطقة غرب المحيط الهادئ، نحن نقدم هنا نظرة متعمقة في كيفية قيام صانعي السياسات الوطنية، والأطراف الخارجية، بدعم الأبعاد المختلفة لتنفيذ سياسة التغذية. إن النظرات المتعمقة الرئيسية لواضعي السياسات الذين يحضرون الاجتماعات التي تركز على الغذاء والتغذية، تتضمن أن تنفيذ سياسة التغذية على مستوى الدولة يعتمد على تصميم سياسات قوي وتخطيط تنظيمي وآليات حوكمة تعزز المسؤولية الجماعية عبر قطاعات متعددة. إن واضعي السياسات المسؤولين عن تنفيذ سياسات التغذية، يواجهون تحديات كبيرة ناتجة عن القدرات المحدودة، سواء داخل الحكومة وخارجها، لا سيما فيما يتعلق بأنشطة الرصد والإنفاذ. سيتطلب التنفيذ الناجح لتدابير سياسة التغذية إرادة سياسية أكبر لتوفير الموارد المطلوبة والهياكل المؤسسية لضمان فعالية السياسة المستدامة. إن الشركاء غير الحكوميين، بما في ذلك الهيئات الدولية والباحثين، لديهم فرصة لدعم تنفيذ السياسات من خلال توفير الدعم الفني للدول الأعضاء للتركيز على الإجراءات الخاصة بالتغذية بطريقة أكثر إقناعًا. كما يمكنهم أن يساعدوا واضعي السياسات في بناء القدرة التنظيمية والهيكلية لتنسيق السياسات عبر القطاعات. يمكن أن يؤدي تحسين تصميم السياسات والتخطيط والحوكمة وبناء الكفاءة الاستراتيجية، بدعم من الشركاء الخارجيين، إلى تعزيز التنفيذ المستدام لسياسة التغذية الشاملة لعدة قطاعات وتحسين نتائج التغذية.

## 摘要

实施有效的跨部门营养政策仍然是全世界的一项挑战。通过查阅世界卫生组织 (WHO) 为代表西太平洋地区各成员国的政策制定者召开的会议和磋商报告,我们深入了解了国家政策制定者和外部参与者如何为实施不同层面的营养政策提供支持。。参加以粮食和营养为要点的会议的政策制定者的主要见解包括,国家层面营养政策的实施依赖于强有力的政策设计、组织规划和治理机制,以促进多部门的集体责任。负责实施营养政策的决策者面临着政府内外能力有限的重大挑战,特别是在监测和执法活动方面。营养政策措施的成功实施将需要更大的政治意愿,以提供必要的资源和体制结构,确保政策的持续有效性。非政府合作伙伴,包括国际机构和研究人员,可通过向成员国提供技术支持来支持政策实施,协助成员国以更加令人信服的方式制定营养行动。还可帮助政策制定者建立协调跨部门政策的组织和结构能力。在外部合作伙伴的支持下,改进政策设计、规划和治理、战略能力建设,可以加强跨部门营养政策的持续实施并改善营养效果。

## Резюме

Реализация эффективной межсекторальной политики в области питания остается сложной задачей, актуальной во всем мире. Изучая отчеты совещаний и консультаций Всемирной организации здравоохранения (ВОЗ), созываемых для политического руководства государств-членов региона западной части Тихого океана, авторы сообщили о том, как лица, определяющие национальную политику, а также внешние субъекты могут поддерживать различные уровни реализации политики в области питания. Ключевые выводы политического руководства, участвующего в совещаниях по вопросам продовольствия и питания, включают в себя то, что реализация политики в области питания на уровне стран опирается на четкую разработку политики, организационное планирование и механизмы управления, которые способствуют коллективной ответственности во многих секторах. Политические деятели, отвечающие за реализацию политики в области питания, сталкиваются с серьезными проблемами, связанными с ограниченными возможностями как самих правительств, так и их окружения, особенно в отношении деятельности по мониторингу и обеспечению соблюдения законов. Успешная реализация мер политики в области питания потребует большей политической воли для предоставления необходимых ресурсов и институциональных структур для обеспечения устойчивой эффективности политики. Неправительственные партнеры, в том числе международные агентства и исследователи, имеют возможность поддержать реализацию политики, предоставляя государствам-членам техническую поддержку для принятия более адекватных мер в области питания. Они также могут помочь политическому руководству в создании организационного и структурного потенциала для координации межсекторальной политики. Улучшение разработки политики, планирования и управления ею, а также наращивание стратегического потенциала при поддержке внешних партнеров может укрепить устойчивую реализацию межсекторальной политики в области питания и улучшить ее результаты.

## Introduction

Since 2015, undernourishment as well as the prevalence of overweight and obesity have increased in many countries.^1^ The burden of malnutrition is a global health priority and Member States of the United Nations (UN) have committed to act during the Global Decade of Action on Nutrition (2016–2025).^2^ Dietary risk factors play a significant role in the development of noncommunicable diseases,^3^ which caused 41 million of the 55 million global deaths in 2019.^4^ Dietary factors are also a key determinant of malnutrition and nutrient deficiencies. Hence, improving population diets could avert one in five deaths globally.^5^ More recently, diet-related noncommunicable diseases have been associated with coronavirus disease 2019 (COVID-19) severity.^6^

The World Health Organization (WHO) has identified strategies for improving diets and health,^7^^,^^8^ and 77% (128) of the 167 WHO Member States surveyed for the Global Nutrition Policy Review (2016–2017) had comprehensive nutrition policies in place to address all forms of malnutrition.^9^ However, the implementation of specific globally recommended policies for addressing diet-related noncommunicable diseases has been uneven.^10^ Policy advocates have faced persistent challenges in developing measures to promote healthy food environments. These challenges include strong opposition from the food industry, pressures to design policies that minimize impacts on trade^11^^–^^13^ and lack of data to convincingly demonstrate the link between particular policies and health outcomes.^14^ Additionally, hard-earned political attention towards diet-related noncommunicable disease prevention may now be displaced by the global response to COVID-19.^15^^,^^16^

Evidence suggests that implementing food and nutrition policy measures adopted by governments is a persistent challenge.^17^^,^^18^ In particular, high reliance on non-health sectors, including education, trade, finance and commerce, to implement many globally recommended nutrition policies has been identified as a common impediment to successful implementation.^19^ Most countries (135/169 surveyed) have adopted governance mechanisms that oversee the implementation of cross-sectoral nutrition policies,^20^ but evidence suggests that these mechanisms often are ineffective at fostering the uptake of nutrition actions across those sectors.^19^^,^^21^ While many influences on policy implementation are specific to the local context, the common challenges underlying these factors, including poor political commitment to nutrition and limited human and financial capacity, suggest there is an opportunity for regional and other cross-country learning.^22^^,^^23^ However, evidence is limited regarding ways to facilitate effective policy implementation.^24^^,^^25^

## Records for policy insights

WHO regularly convenes regional meetings and consultations with Member State representatives to discuss and consult on key policy challenges facing countries, to assess country progress, and to facilitate cross-regional communication on the implementation of effective policy measures. Food and nutrition policy-makers working at national and regional levels have a collective experience on factors that promote or inhibit the scaling up and sustained implementation of cross-sectoral nutrition policies. Their perspectives and experiences are representative of their communities of practice^26^ and their learned experience can inform practice-based strategies.^27^ Hence, reports from these meetings can be used to assess policy-makers’ insights and lessons learnt from the implementation of cross-sectoral nutrition policy measures. 

To identify strategies that can sustain the implementation of cross-sectoral food and nutrition policies, we drew information from the meeting reports of all food or nutrition-centred meetings and consultations hosted by the WHO Regional Office for the Western Pacific. In the Western Pacific Region, noncommunicable diseases cause an estimated 80% of all deaths,^28^^,^^29^ underscoring the need for preventive policy action. Member States in the region have collectively taken action to adopt WHO-recommended food and nutrition policy measures^9^^,^^29^^,^^30^ and, thus, policy-makers have a wealth of experience navigating nutrition policy implementation.^13^^,^^31^^–^^33^

**Fig. 1:**
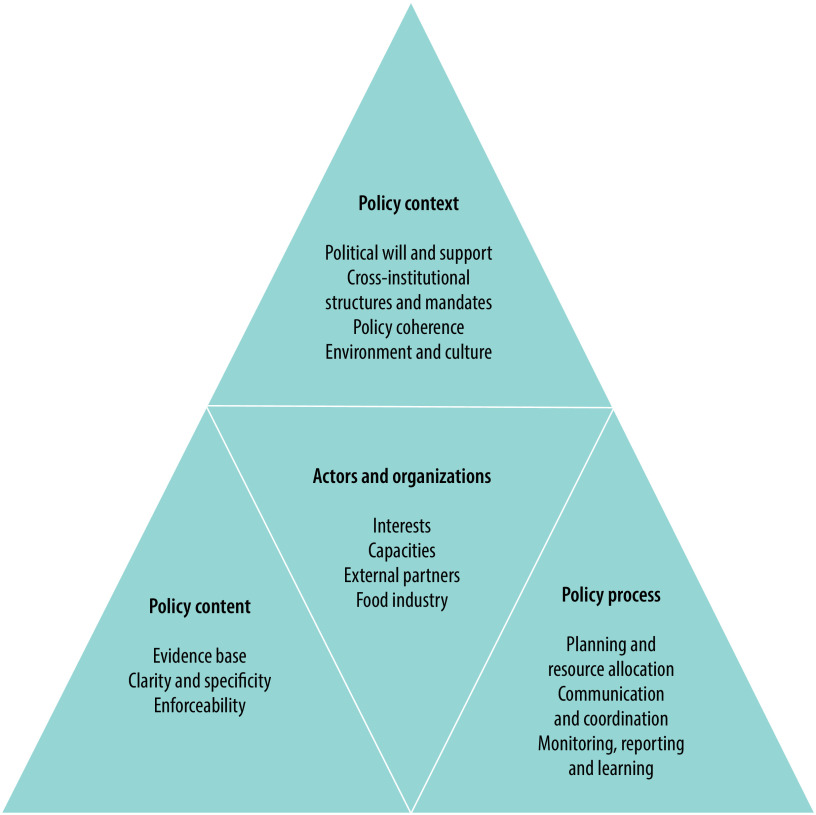
Analytic framework used for analysis of meeting reports from nutrition-related consultations and meetings, Western Pacific Region, 2004–2020

Source: Adapted from Walt & Gilson.^35^

We retrieved meeting reports from WHO’s Institutional Repository for Information Sharing^34^ for meetings held between the launch of the Global Strategy on Diet, Physical Activity and Health in 2004 and the end of 2020. We included biregional or subregional meeting reports, drawing on text reported as participant presentations, groupwork or panel discussions led by Member State representatives. One author extracted all data from the included reports while a second author, who has a background in policy implementation, verified all extractions. We analysed the content of the meeting reports using a deductive coding framework based on well established theories of policy implementation and capacity needs (Fig. 1).^35^^,^^36^ The coding framework included codes related to: (i) the policy process (that is, policy context, process, content and actors);^35^ (ii) institutional structures and policy implementation;^37^^–^^39^ and (iii) systemic capacity needs, particularly in relation to organizational systems (e.g. structures, systems, supervision, role clarity, decentralized power, planning systems).^36^

Between 2004 and 2020, WHO Regional Office for the Western Pacific hosted 29 food and nutrition-centred meetings, consultations and workshops (Table 1). From 2012, we observed a shift from meetings focused on single nutritional risks (e.g. anaemia, salt) towards solution-oriented meetings, directed at noncommunicable disease prevention. Of the meetings, eight related to food safety, seven were related to restrictions on food and beverage marketing and the others largely related to nutrition in early childhood, nutrition surveillance or policy tools that support planning and implementation.

**Table 1:** Member State nutrition-related consultations and meetings, Western Pacific Region, 2004–2019

Meeting	Year	Location	No. of Member States in attendance
Future action on food safety in the Pacific: FAO/SPC/WHO meeting	2004	Seremban, Negeri Sembilan, Malaysia	14
Asia-Pacific workshop on raising the profile of nutrition	2007	Kuala Lumpur, Malaysia	7
WHO/UNICEF consultation on breastfeeding protection, promotion and support	2007	Manila, Philippines	16
FAO/WHO meeting on food standards to promote health and fair trade in the Pacific	2008	Manila, Philippines	13
Regional consultation on strategies to reduce salt intake	2010	Singapore	11
Anaemia prevention along the life-cycle	2010	Hanoi, Viet Nam	2
Prepare a draft Western Pacific Regional Food Safety Strategy 2011–2015 for consideration of the Regional Committee for the Western Pacific	2010–2011	Manila, Philippines and Selangor, Malaysia	24
Pacific Food Summit	2010	Port Vila, Vanuatu	20
Technical consultation on identifying approaches to control obesity	2011	Melbourne, Australia	10
Meeting on regulatory monitoring of salt and flour fortification programmes in Asia	2011	Manila, Philippines	9
Japan-WHO regional consultation for promoting healthier dietary options for children	2012	Saitama, Japan	12
Informal consultation on reducing the harmful impact on children of marketing foods, beverages, tobacco and alcohol	2013	Manila, Philippines	7
Meeting on strengthening INFOSAN and national food control systems in Asia	2013	Manila, Philippines	11
Consultation on the Action Plan to Reduce the Double Burden of Malnutrition in the Western Pacific Region, 2015–2020	2013	Manila, Philippines	14
Consultation on overweight, obesity, diabetes and law in the Western Pacific Region	2014	Manila, Philippines	14
Fifth international workshop on total diet studies	2015	Seoul, Republic of Korea	6
Technical meeting on the regional adaptation of the WHO nutrient profile model to the Western Pacific Region	2015	Manila, Philippines	7
Pacific workshop on nutrition, noncommunicable diseases and the role of Codex	2015	Nadi, Fiji	10
Meeting on strengthening the international food safety authorities network (INFOSAN) in Asia and national food safety systems	2015	Hong Kong Special Administrative Region, China	21
Biregional workshop on restricting the marketing of foods and non-alcoholic beverages to children in the Western Pacific and South-East Asia	2015	Kuala Lumpur, Malaysia	13
Workshop on implementation and monitoring of the Action Plan to Reduce the Double Burden of Malnutrition in the Western Pacific Region (2015–2020)	2015	Manila, Philippines	10
Informal consultation on childhood obesity surveillance in the Western Pacific Region	2016	Tokyo, Japan	11
Regional workshop on regulating the marketing and sale of foods and non-alcoholic beverages at schools	2016	Manila, Philippines	15
Technical workshop on taxing sugar-sweetened beverages	2016	Manila, Philippines	7
Pacific consultation on the draft Regional Framework for Action on food safety in the Western Pacific	2017	Nadi, Fiji	18
Experts consultation on the background document on protecting children from the harmful impact of food marketing	2017	Manila, Philippines	7
Consultation on the draft Regional Framework for Action on Food Safety in the Western Pacific	2017	Manila, Philippines	12
Experts consultation to inform the development of a draft Regional Action Framework on Protecting Children from the Harmful Impact of Food Marketing: 2020–2030	2018	Manila, Philippines	6
Member States consultation on the Regional Action Framework on Protecting Children from the Harmful Impact of Food Marketing: 2020–2030	2019	Manila, Philippines	22

FAO: Food and Agriculture Organization; SPC: Pacific Community; UNICEF: United Nations Children's Emergency Fund; WHO: World Health Organization.

Note: Reports are available from WHO Institutional Repository for Information Sharing.^34^

## Dimensions of policy implementation

Through the meeting records, we found that contributions by policy-makers at meetings largely centred on four dimensions of policy implementation: (i) the implementation of specific WHO-recommended policy measures to promote improved nutrition; (ii) systems for monitoring and learning from implementation; (iii) the coordination of institutional and governance structures that facilitate engagement of multisectoral government actors; and (iv) the implementation of mechanisms to manage engagement with external stakeholders in nutrition policy (Table 2). Examples of contributions are given in the data repository.^40^

**Table 2:** Perspectives of Member State representatives on enablers of nutrition policy implementation, extracted from relevant Western Pacific Region of the World Health Organization meeting reports 2004–2019

Dimension of implementation	Themes	No. of meeting reports supporting theme
Implementing specific policy measures	Human and financial capacity	18
	Strong policy design	23
	Clear systems, structures and roles	14
	Building support for policies	13
Implementing systems for monitoring and learning	Monitoring mechanisms	12
	Effective reporting and learning	10
Implementing governance structures that engage multisectoral government actors	Mechanics of governance	16
	Maintaining political support	19
Implementing processes to manage engagement with external stakeholders	Harness the commitment of external stakeholders	14
	Monitor industry influence and manage conflicts of interest	14

### Implementing policy measures

The need for improved or strengthened implementation of policy measures was discussed at 27 meetings. Policy-makers identified a range of challenges to implementing specific nutrition policy measures recommended by WHO, which often related to difficulties in communicating and collaborating with implementing stakeholders and the lack of support from decision-makers in actioning the necessary policy measures. For example, policy-makers often reported being unable to access the requisite funds or personnel to promote and enforce policy activities on an ongoing basis. Policy-makers also frequently mentioned that unclear policy content could undermine a strong and effective policy response. For instance, nutrition regulations commonly lacked clear definitions and ways to classify the healthiness of food or they did not identify mechanisms for enforcement, such as sanctions.

Meeting participants clearly articulated the systemic capacities they would need to better implement nutrition policies in their countries. For instance, policy-makers wanted clearer operational plans for implementing specific policies that identify roles and responsibilities of implementing stakeholders, and clearer oversight and supervision from senior leadership. They identified greater policy coherence as a strategy for overcoming capacity limitations. For example, they called for changes to food regulations to ensure food labelling mandates contain requisite information to support a strong policy response and the integration of nutrition activities into the operational plans and responsibilities of a broader number of stakeholders. Policy-makers also called for nutrition policies to be underpinned by clear definitions to enable straightforward interpretation and implementation, and strong and clear sanctions to enable effective enforcement. 

Policy-makers identified the need for a more consistent, compelling and contextually relevant body of evidence demonstrating policy effectiveness to convince governments to commit the requisite resourcing towards nutrition policy implementation and provide appropriate oversight. They also expressed the need for coherent evidence to inform decisions around specific policy parameters (Box 1): for example, consistency in definitions of healthy and unhealthy foods and ages of children to be targeted by restrictions on food marketing.

Box 1 **Research requested to support country-level implementation of nutrition policies, Western Pacific Region, 2020**

- Robust, contextually relevant evidence demonstrating economic and social gains associated with policy measures, including the impact of inaction
- Development of policy briefs that anticipate and, where applicable, decisively counter non-evidence based industry opposition to globally recommended policy measures
- Development of clear technical standards and definitions around policy inclusions and targets, for example, definitions on unhealthy foods, effective rates for food taxes and age of children to be targeted by marketing restrictions
- Information on investment levels and support requirements for implementation of different policies
- Research on implementation effectiveness and contextual influences on policy processes
- Multicountry comparison on the effectiveness of various policy approaches
- Development of standardized monitoring and surveillance frameworks for priority policies
- Evidence to improve the functionality of multisectoral coordination mechanisms
- Clarity of alignment between nutrition and other development or economic goals (e.g. SDGs, child protection, poverty and gender issues)

SDGs: sustainable development goals.Note: We extracted policy-makers’ perspectives from reports of Member State consultations and meetings focusing on food and nutrition.

### Monitoring and learning

Opportunities to strengthen the implementation of systems for monitoring and learning were discussed at 21 meetings. Policy-makers indicated they were not confident in systems for monitoring and reporting in their countries, in particular noting that data were not being reported back in a way that would necessarily incite reorientation or strengthening of policy measures. They reported lacking the capacity and tools for data collection and reporting, especially where other ministries and stakeholders were involved.

The ability to demonstrate the impact of successful measures was identified as a key determinant for maintaining accountability and garnering political and cross-sectoral support. In the context of capacity constraints, policy-makers identified the critical need for more efficient and reliable mechanisms to collect and collate routine information on policy implementation and compliance (Table 2). Additionally, policy-makers called for monitoring efforts to be extended beyond epidemiological indicators to include contextual influences on policy process and implementation effectiveness.

Meeting participants also noted that policy lessons should be presented such that they are easily interpreted and acted on by those with the power to influence political or public opinion (e.g. experts, media, civil society advocacy groups and community leaders). In order to more powerfully convey relevant nutrition policy messages, policy-makers were seeking skills in knowledge translation.

### Multisectoral governance structures

In the reports, there was substantial policy-maker dialogue on mechanisms to strengthen multisectoral coordination and cooperation (17 out of 29 meetings). While most countries had interagency coordination mechanisms to facilitate multisectoral action, these were not necessarily facilitating meaningful dialogue or action. Of primary concern for policy-makers was the perceived lack of priority for nutrition among non-health actors within government, and associated difficulties mobilizing and sustaining input from them over time. Low political will for nutrition measures across government and among political leaders was widely reported at meetings.

Cross-ministerial championship from leaders was deemed critical for eliciting support across sectors (Table 2). In particular, policy-makers believed decision-makers needed to facilitate a cross-sectoral dialogue that reflects on both problems and solutions. Policy-makers from Member States that had achieved some level of policy success attributed that success to the oversight and championship of political leaders. Some of the mechanisms believed to be helpful in inciting and maintaining political interest in nutrition were: the availability of accurate, compelling and (where possible) nuanced data demonstrating the need for the particular policy approach; clear demonstration of both cost–effectiveness and the investment required to achieve policy effectiveness; alignment of policy measures with international accountability systems and commitments; and availability of contextually relevant information demonstrating feasibility and benefits across multiple government sectors.

### Managing external stakeholders

Engagement with influential external stakeholders in the nutrition policy space was discussed at 21 of the 29 meetings. External stakeholders most often referred to were regional development partners (including the UN, donors and regional technical support programmes), civil society groups and the food industry.

Policy-makers called for stronger regional and bilateral cooperation to amplify nutrition policy efforts, especially in relation to food regulation, trade and cross-border sales and marketing. Meeting participants considered regional organizations to be in the best position to facilitate functional relationships across countries, for instance, by developing specialist support networks and actively negotiating agreements across common policy challenges. WHO, in particular, was called on to oversee greater policy coherence for nutrition by UN partners working at the country-level and to oversee a research agenda for nutrition policy. Several information gaps and research priorities were identified by policy-makers attending the meetings (Box 1).

Policy-makers frequently referred to the value of a strong and well informed civil society to engage on food and nutrition challenges. However, they noted that civil society groups often lacked the technical capacity and resources for credible and meaningful engagement and they requested that development partners work with these groups to develop the necessary strategic and technical capacities.

Meeting discussions raised numerous examples of where the food industry had undermined or weakened policy implementation efforts, by questioning the mandate of the enforcing agency, by legally opposing or circumnavigating policy rules or by targeting policy implementers (e.g. teachers and nurses). The policy-makers called on academia to enhance their preparedness for diminishing industry influence, for example, by providing evidence to persuasively counter industry objections to globally recommended policies and by developing systems to monitor food industry activity and expose violations of policy measures.

## Way forward

Using the findings from our review and with reference to the published literature, we discuss below opportunities to improve policy design, planning, governance and implementation of globally recommended nutrition policies.

### Capacity for implementation

Consistent with our theory on capacity,^36^ we identified that limitations related to a lack of staffing and resourcing need to be considered during policy development. Capacity challenges could also be partly alleviated through building the systemic capacity of implementing agencies and civil society partners,^36^^,^^41^^,^^42^ that is, the broader strategic and operational capacities required to fully implement a nutrition policy. For instance, skills in planning, implementation and actor mobilization,^41^^,^^43^ and confidence to generate and interpret monitoring and research for learning and accountability,^41^^,^^43^ are all likely to be vital for action on nutrition. This approach will require a reorientation of capacity development beyond a focus only on knowledge and technical skills, and towards more strategic competencies including coordination, negotiation, advocacy and leadership.^41^^–^^43^ The findings are also consistent with analyses of national nutrition policies that have found that policies often lack the requisite technical detail and mandate to facilitate a strong and effective policy response.^17^^,^^41^

### Governance and political support

Consistent with previous research, the meeting reports confirm that political rhetoric for nutrition is, in many cases, unmatched at the country level by the resources and institutional structures necessary to institute strong policies and to sustain long-term nutrition gains.^22^^,^^44^^,^^45^ Lack of political interest in nutrition is a key reason to why policy implementation is typically insufficiently resourced, and the lack of impact resulting from under-resourcing further compromises commitment.^44^^,^^45^

Given challenges associated with mobilizing cross-sectoral actors, attracting cross-ministerial oversight of policies affecting population nutrition is needed. Such oversight should facilitate productive dialogue between cross-sectoral leaders and maintain their accountability for addressing nutrition issues.^14^^,^^41^^,^^45^

Implementation of recommended policies for improving nutrition is therefore reliant on garnering the support of policy leaders to engage across all dimensions of policy implementation. To elicit their interest, it will be vital to build the collective capacity of nutrition policy-makers and advocates to act in unison to position nutrition as a matter of importance across several sectors.^46^ For example, policy-makers could better position nutrition as an economic and development imperative for action across several public areas, such as human rights and educational outcomes. This framing may be critical in settings where powerful food industry interests undermine and resist increased regulations related to nutrition.^47^

### External partners

The insights from this review support the operationalization of calls for greater political commitment to improving nutrition.^14^^,^^44^^,^^45^ External partners play an important role in generating political will by lending legitimacy to nutrition issues.^45^^,^^46^ Regional development partners can strengthen systemic and organizational capacity in countries,^36^^,^^41^ and can convene bilateral and regional actor networks to amplify collective efforts across countries.^45^^,^^47^ Furthermore, regional development partners should review their portfolios of support across multiple sectors to ensure that they are promoting coherence with nutrition policies.^48^

Our review suggests that WHO meetings can play an important role by engaging policy leaders in dialogue on what is required to sustain effective implementation beyond policy adoption. WHO already encourages research partners to contribute to evidence-based policy development and facilitates several global accountability mechanisms relevant to nutrition, including the *Global nutrition policy reviews*.^49^ However, WHO has an opportunity to encourage researcher partners to contribute further to the development of reliable and efficient policy monitoring and accountability systems.

WHO has established several region-specific nutrient profiling models to strengthen policy implementation, but this analysis of policy-maker perspectives suggests that expanding technical guidance to include further best-practice standards for nutrition policies is needed: for example, sugar-sweetened beverage tax design (that is, effective tax rates and taxation mechanisms) and clear definitions for restrictions on marketing to children. A more cohesive approach to best-practice nutrition policy guidance may be of great practical value for countries, by fostering consistency and efficiency, and strengthening justifications underpinning policy parameters.^50^ WHO’s Framework Convention for Tobacco Control (FCTC) offers a precedent that could also guide nutrition policy. The comprehensive policy agenda of FCTC requires approval by high-level leaders and whole-of-government action. Furthermore, the FCTC’s strong and consistent accountability mechanisms have weakened grounds for opposition by industry.^47^

### Meeting reports as a data source 

The meeting reports we analysed proved useful in capturing aggregated contributions and views of representing officials while overcoming sensitivities commonly associated with interviewing senior policy officials.^51^ The findings were consistent with more intensive approaches to data collection (e.g. in-depth interviews and review syntheses).^41^^,^^42^^,^^45^ In our case, aggregating the meeting reports represented an efficient mechanism for gathering the perspectives of policy-makers working across a range of diverse policy settings.

However, we acknowledge that the meeting reports represent summaries of proceedings by rapporteurs, rather than a verbatim record of proceedings, and are variable in the level of detail included. As an aggregated source of information, the individual views of policy-makers are rarely made explicit as part of the meeting reports. Further, the contributions of meeting participants are likely to reflect the perspectives of attending individuals and, as such, the diversity of opinions from Member States is not likely to be fully represented. The meeting reports are subject to reporting bias and should be interpreted with caution. However, the breadth of representation from Member States at meetings, and the relevance of the meetings to nutrition policy somewhat countered these limitations. While the meeting reports represent only views from the Western Pacific Region, our comparison with nutrition policy research conducted in other regions^41^^,^^42^ and globally^44^^,^^45^ has elucidated that the challenges being reported are similar to those being experienced in other contexts, and the lessons are therefore likely to be applicable to other regions. Nevertheless, the relevance of the findings to other regions requires further examination.

## Conclusion

Our review revealed that policy-makers coordinating nutrition policy development and implementation within countries face challenges when implementing such policies, particularly in relation to monitoring and enforcement activities. These challenges result from a lack of human and financial resources across agencies that is required to support implementation. In many settings, governance mechanisms provide inadequate oversight to the commitments and responsibilities health and non-health actors have towards nutrition policies. The lack of commitment to addressing nutrition issues across government likely undermines critical aspects of policy implementation within countries. However, policy-makers have identified key enablers of sustained nutrition policy implementation, and call on political leaders and external partners to support them in progressing towards achieving successful implementation of best-practice food and nutrition policy measures.
